# Spontaneous action matching in dog puppies, kittens and wolf pups

**DOI:** 10.1038/s41598-023-28959-5

**Published:** 2023-02-16

**Authors:** Claudia Fugazza, Andrea Temesi, Roberta Coronas, Stefania Uccheddu, Márta Gácsi, Ákos Pogány

**Affiliations:** 1 Department of Ethology, Eötvös Loránd University, Budapest, Hungary; 2 ELTE NAP Comparative Ethology Research Group, Budapest, Hungary; 3 MTA-ELTE Comparative Ethology Research Group, Budapest, Hungary

**Keywords:** Evolution, Psychology

## Abstract

We investigated the spontaneous tendency of dog puppies, kittens and wolf pups to match their behaviour to actions demonstrated by a human, in the absence of food reward. Based on dogs’ inherent sociality and domestication history, we predicted that the tendency to match human actions is more pronounced in this species than in the other two. To test this, we exposed N = 42 dog puppies, N = 39 kittens and N = 8 wolf pups to ostensive human demonstrations of an object-related action. We found that dog puppies paid more attention to the demonstration than kittens and wolf pups. Dog puppies and wolf pups matched the demonstrated actions in more trials than kittens. Dog puppies also tended to reproduce the human demonstration that differed from the action they typically preformed in the absence of demonstration. These results support that dog puppies show a tendency to attend to humans and conform their behaviour to human demonstrations in the absence of extrinsic food rewards. This spontaneous tendency is also relevant for practical applications, by providing the basis to devise puppy-training methods that rely less on food rewards, and instead exploit puppies’ natural predisposition for social learning.

## Introduction

Young individuals of species with prolonged parental care may be inclined for replicating the actions of their parents or other group members, as social learning largely impacts their fitness by reducing the costs of individual learning^1,2^. Moreover, early learning through observation may improve the development of their skills (e.g.^3^).

In social species, the tendency to match actions of other group members may not only serve a learning function, but it may also facilitate social cohesion and affiliation^4,5^. In humans, the tendency to match actions in the absence of an immediate goal or extrinsic reward is well known (e.g.^4^) and there is some evidence also for non-human primates^5^. However, social learning studies on non-human species mostly include tasks in which the subjects are motivated by food, or where reproducing some aspects of the demonstration is aimed at reaching a reward (e.g.^6–9^).

A large body of empirical data has been gathered on adult dogs’ and, more recently, also on dog puppies’ ability to learn socially^10^. Dogs are known for their flexibility in learning not only from conspecific (e.g.^11^), but also from human demonstrators (e.g.^6,12^).

Interestingly, in a study on overimitation^13^, dogs even showed a tendency to reproduce an irrelevant action - i.e. an action that did not lead to the food reward. However, since food was used to motivate the subjects before the demonstration, it is not known whether the subjects would have reproduced their owners’ actions in the absence of food.

Another domesticated species that lives in close contact with humans is the domestic cat (*Felis catus)*. This places cats, along with dogs, among the few especially interesting species that have ample opportunity for social learning from humans. Despite an increased research interest in understanding cats’ behaviour and cognition (e.g.^14–16^), controlled studies on their social learning skills are almost completely lacking. Fugazza et al.^8^ provided limited evidence on a single cat and, also in this case, food incentives and rewards were used to train the cat.

Dogs’ and cats’ evolutionary history differ in three important aspects, which may affect their tendency to interact socially with humans. First, dogs and cats are different in their initial, ancestral social systems^17,18^. Cat’s ancestors (*Felis silvestris lybica*) were solitary hunters, while dogs’ closest relatives were pack hunters with a tendency toward scavenging. Secondly, the divergence between the dog and modern wolves took place between 20,000 and 40,000 years ago^19^. Cats, in contrast, were domesticated more recently, approximately 10,000 years ago^20^. Thirdly, during their history in the human environment, dogs often fulfilled cooperative functions, whereas cats were primarily kept because their typical preys included rodent pests^20^.

Different selective pressures and different duration of domestication might have resulted in distinct predispositions towards social learning when interacting with humans. Wolves are the living species that is closest to dogs’ ancestors. Thus, studying the similarities and differences between young dogs, cats and wolves that were raised similarly in the human environment, may contribute to shedding light on (1) the role of the inherent social or solitary nature that should be apparent in the ancestral species, leading to the hypothesis of enhanced social learning in the more social species (dog and wolf) compared to the one with solitary origins (cat); (2) the process of domestication, leading to the hypothesis of enhanced social learning tendencies when interacting with humans in the domesticated species (dog and cat), compared to the non-domesticated one (wolf) and (3) different domestication histories, leading to the hypothesis of enhanced social learning in the species domesticated for cooperative functions (dog), compared to the one domesticated mostly for independent hunting (cat).

Several studies compared socialized wolves and dogs to reveal the effect of domestication on the behavioural predispositions of dogs, particularly in the socio-cognitive domain. However, comparative studies on social learning in dogs and hand-raised wolves, especially when learning from humans (e.g.^21^), are scarce. Range and Virányi^9^ found that both dogs and wolves benefitted from human and conspecific demonstrations in a local enhancement task involving the hiding of a food reward. In a different study, the same authors reported that wolves outperformed dogs in learning a manipulative task from a conspecific. Unfortunately, in this study human demonstrations were not included^22^.

We aimed at testing the tendency to *spontaneously* reproduce actions demonstrated by humans in comparably socialized dog puppies, kittens and wolf pups, all living in human families. By *spontaneous,* we refer to actions replicated in the absence of extrinsic reward (food) and in the absence of previous training.

We first assessed what actions the subjects typically performed when they had the possibility to interact with a novel object (a box or a Wobbler Kong), in the absence of a demonstration. To test whether a preference for an action (i.e. a particular way of interacting with the object) could be reversed by demonstrating a different behaviour, actions performed in the absence of any demonstration were contrasted with the ones performed after the demonstration of a different behaviour. The tendency to match the demonstrated (non-typical) action would thus show a predisposition for action matching even in the absence of food reward.

To test action matching, we relied on the two-action method, which had previously been successfully applied to test imitative abilities in various species, including dogs^23–25^.

Since previous studies highlighted the importance of ostensive cues in social learning situations in dogs^26^, we included ostensive cues in our demonstrations.

Depending on the definition used by different authors, the execution of a motor action after its observation is called imitation (e.g.^27^) or response facilitation^28,29^. Imitation is typically considered to account for behavioural similarity when the imitated action is novel or otherwise improbable^30^. Based on this approach, since we first determine the typical action, and then contrast it with a different demonstrated one, it could be argued that performing the same (non-typical) action, in response to a demonstration, could be considered as imitation. However, since we included only one “improbable” action, we prefer to refrain from giving a specific label to the observed behavioural similarity, and instead use the more operational term “action matching”.

Considering that all the subjects of this study were raised by humans and experienced intensive social interactions with them while living in human families, it could be hypothesized that young subjects all 3 species may show similar predispositions to match their actions to humans’ in the absence of food rewards. However, we hypothesized that the extent to which this tendency manifests itself may be related to the species’ inherent social or solitary nature and their evolutionary history. Thus, based on the inherent cooperative nature of dogs and wolves, we predicted that dog puppies’ and wolf pups’ behaviour would be more affected by the human demonstrations, than kittens. Based domestication history of dogs and cats, we also predicted that dog puppies would show a greater tendency to attend to human demonstrations and to spontaneously match their actions to those of humans, compared to kittens.

## Methods

### Ethical permit

Ethical permission for conducting this study was obtained from The Institutional Committee of Eötvös Loránd University (N. PE/EA/692-5/2019). All experiments were carried out in accordance with relevant guidelines and regulations for both owners as volunteers and their animals participating in the experiments. Owners gave informed consent to participate in the study with their animals. The study is reported in accordance with ARRIVE guidelines. Informed consent was obtained to publish images in an online open-access publication.

### Subjects

We tested 42 dog puppies of various breeds (mean ± SD age = 13.4 ± 1.7 weeks old), 39 kittens (13.8 ± 2.6 weeks old) and 8 wolf pups (12 ± 2.8 weeks old), 5 of which belonged to the same litter. All subjects of all species were socialized, lived in human families, and experienced the typical life of family companion animals. The wolves were hand-raised by their caregivers after being separated from their mothers in the first 12 days from birth, independently from the present study. They were bottle-fed and like the dog puppies and kittens, lived in the owner’s family, thus were in close contact with them during their early development (3 wolf pups were raised and lived in human families in Mexico, were registered by their breeders and were kept according to the regulations of the Secretaría de Medio Ambiente y Recursos Naturales; 5 wolf pups originated from the Horkai Animal Training Center at Gödöllő, Hungary, and were raised in human families in Hungary. For further details, see Ujfalussy et al.^31^). Living in their human families, they regularly met strangers and conspecifics, similarly to the other two species included in this study.

### Testing site

Depending on the owners’ availability, the tests were carried out at the Department of Ethology at Eötvös Loránd University, or at the owner’s home (subjects tested at the Department: N = 32 of 42 dog puppies; N = 20 of 39 kittens; and N = 5 of 8 wolf pups). The Experimenters (C.F., A.T., S.U.) carried out all the tests, both at the Department and at the owners’ homes.

### Objects used for testing (Fig. 1)

**Figure 1:**
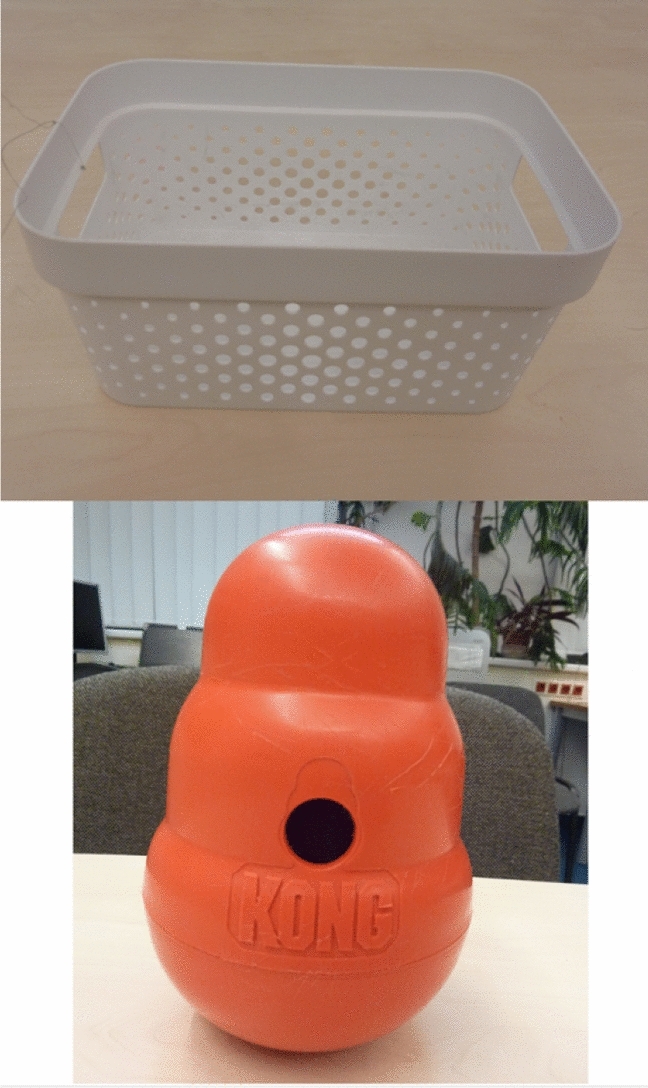
Objects used for testing (Box at the top, Wobbler Kong at the bottom).

Two unfamiliar objects were used: (1) a white plastic box (ca. 12 × 26 × 17 cm); and (2) a Wobbler Kong (19 × 12 cm). We ensured that the objects were unfamiliar to the subjects by asking the owners if they might have had any previous experience with them and whether they had these objects at home. The objects were allocated randomly to subjects. The same object was used in all trials for a given subject.

### Protocol

Before the start of the tests, the subjects were allowed to get acquainted with the demonstrator, while the test procedure was explained to the owners. The subjects were free in the experimental room (or in the designated test area when tested at home), while the experimenter and the owner talked and briefly greeted the subject when approached. This familiarization lasted until the explanation of the protocol was over (ca. 10 min).

### Exploration trial

Before receiving trials with demonstrations (see below), the subjects were allowed to explore the target object. The aim of the free exploration trial was to observe the subjects’ spontaneous behaviour in the absence of a demonstration and served also as a control for social facilitation, i.e. to exclude the effect of the mere presence of the demonstrator as an explanation for action matching^32^.

At the beginning of the free exploration trial, the owner held the subject on the floor by gently holding it at its chest, at 1.3 m from the target object. The experimenter crouched down within reaching distance from the object and ignored the subject by looking down at her/his knees. The owner released the subject and allowed it to explore for 25 seconds. After the subject was released, we monitored and noted whether the subject touched the object and observed the body part (nose or paw) used for this interaction.

### Trials with demonstrations

Immediately after the free exploration trial, subjects were exposed to trials, which included a demonstration made by a human experimenter.

**Figure 2:**
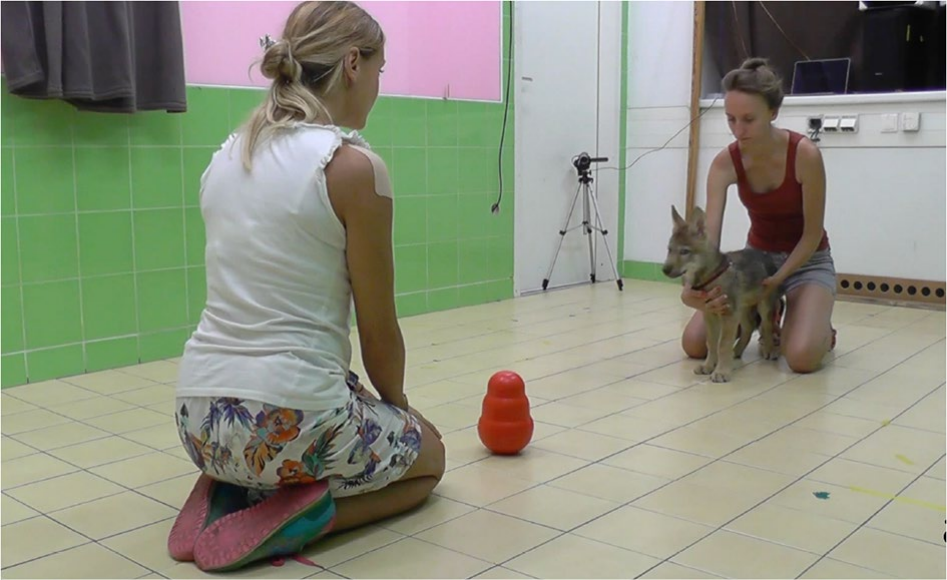
Experimental setup. In the free exploration trial and in the experimental trials, the demonstrator is within reaching distance from the object. The owner gently holds the subject at its chest, in front of him/her. In the trials with demonstration, the demonstrator makes eye contact and uses ostensive communication before and during the demonstration.

In these trials, a pre-determined action was demonstrated (Fig. 2). We used two actions for the demonstrations: touching the object with hand or touching the object with nose. To minimize the possibility of our tests becoming confounded by typical actions, the action that the subjects had eventually performed during the previous free exploration trial was contrasted by the different action demonstrated in the experimental trials. For instance, if the subject touched the object with its nose during the free exploration trial, then the demonstrated action in the forthcoming experimental trials was planned as a hand action and vice versa. If the subject had not interacted with the object during the free exploration trial, then the action to be demonstrated in the following experimental trials was randomly determined (either nose or hand).

The subject was held by the owner similar to the free exploration trial (Fig. 2). The experimenter was kneeling in front of the target object, within reaching distance from it. She first looked at the subject and called its attention by pronouncing its name and/or using attention grabbing vocal signal (e.g. smacking her lips).

As soon as the subject looked at her, the experimenter performed the demonstration. In the case of nose demonstration, the experimenter touched the object with her nose two times. In the case of the hand demonstration, the experimenter touched the object with her hand two times. The experimenter provided ostensive cues also while performing the demonstration by alternating her gaze between the subject and target object and saying sentences like “Look at what I am doing!”, “This is so nice!”. After demonstration, the demonstrator looked down and ignored the subject.

The subject was released by the owner and was free to explore for 25 seconds, or until it interacted with the target object. If the subject interacted with the object, the trial was stopped and the owner gently called the subject back to the starting position for the next trial, while briefly verbally praising it.

In the following two trials, the same procedure was repeated by the experimenter who demonstrated the same action on the same object.

After these 3 trials, the subjects received a short break (approximately 5 to 10 minutes) during which they were taken out of the experimental room, and were then tested in another 3 similar experimental trials with the same target object. The only difference was the movement of the object during the demonstration: in “move” trials, the demonstration resulted in moving the object, while in “touch” trials the object did not move. Order of move and touch trials was randomized between the first and the second set of three repeated trials.

We also carried out three ‘Ghost trials’ in which the object was moved remotely by using a transparent fishing line. This was done to observe whether the movement of the object attracted the subjects’ attention and stimulated interaction with it. Subjects were randomly allocated to this condition or to the experimental one, unless owners were available for repeated testing, in which case subjects were tested in all conditions.

### Data collection and analysis

Experimental trials were video recorded. From the recordings, the following behavioural variables were coded, using Solomon Coder (beta 15.03.15, © András Péter):

- Latency to looking at the demonstration: time measured from the start of the trial, until the head of the subject was oriented towards the object;
- Latency to interacting with the object: time measured from when the subject was released, until it physically interacted with the object using either nose or paw;
- Body part used to interact with the object (binary response for nose and paw, separately): Nose, if the subject interacted with the object by touching it with nose; Paw, if the subject interacted with the object by touching it with paw; Both paw and nose if they touched the object with their nose and paw at the same time.

From the above coded variables, we then derived Action matching (a binary response), defined as the subject interacting with the object using its paw when the demonstration was performed with hand/paw and interacting with the object using nose when the demonstration was performed with nose, including the cases when both paw and nose were used at the same time.

### Statistical analyses

We used the R statistical environment (v. 4.1.2; R Core Team 2021) to analyze our data. For each trial, we coded whether and how fast a given behaviour occurred. Latencies to looking at the demonstration and interacting with the object (separate response variables), were analyzed in Cox Mixed Models (‘CMM’, R package ‘coxme’^33^). In CMMs, time (in seconds) spent until the focal behaviour was performed (i.e. until looking at the demonstration or interacting with the object) was the response, and the focal behaviour was the terminal event. Individuals that did not perform the focal behaviour were treated as censored observations. Probability of action matching, body part used during free exploration and paw use (separate binary response variables) were analyzed using binomial Generalized Linear Mixed Models (‘GLMM’ henceforth, R package ‘lme4’^34^). The analysis of paw use was based on our analysis of the body part used by the subjects when interacting with the object in the free exploration trial; this showed that they almost invariably interacted with the object spontaneously with their nose. Therefore, in models of paw use we analyzed whether the demonstration of a hand/paw action in experimental trials resulted in an increase in paw actions (thus, in contrast to their preference; see ‘increase in paw use’ below).

Both in GLMMs and CMMs, the following fixed effects were analyzed:

- species (factor with 3 levels)
- trial number (factor with 3 levels)
- condition (factor with two levels: ghost or experimental)
- testing site (factor with two levels: department or home)
- touch or push (factor with two levels: ‘touch’: the demonstration did not result in object movement or ‘push’: the demonstration resulted in object movement)
- body action used for demonstration (factor with two levels: nose or hand – included only in models of paw use).

In addition to the above fixed effects, all models included subject ID as a random effect. The effects of explanatory variables were analyzed by likelihood ratio tests, and non-significant explanatory variables were excluded from the final models based on a stepwise model selection. We also provide parameter estimates (for GLMMs) and hazard ratios (exp[β], with 95% CI, for CMMs) between levels of a given significant fixed factor.

The final models of interacting with object included both trial and touch or push (see “Results”). The latter variable, however, had no value for the free exploration trial, hence we could not investigate whether subjects behaved differently in trials that were preceded by a demonstration as opposed to the free exploration trial. Therefore, for interaction with object, we also carried out separate analyses for each species, focusing only on the effect of trial.

We applied the same approach for analyzing paw use; following the analysis including all three species that was limited by low occurrence of interacting with the object in kittens, we also carried out separate analyses for each species to reveal more details for dogs and wolves.

## Results

### Looking at demonstration

**Figure 3:**
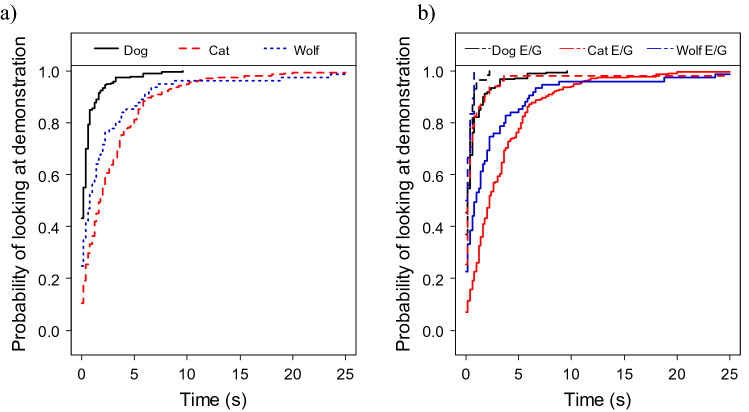
(**a**) Species differences: dog puppies were more likely to look at the demonstration than wolves and kittens and (**b**) species-specific effects of condition (experimental condition, E, illustrated with solid, and ghost condition, G, with dotted line) on the probability of looking at the demonstration: dog puppies looked at the demonstration in both, experimental and ghost trials; kittens and wolves looked more at the demonstration in the ghost trials than in the experimental trials, with this difference being more pronounced in kittens.

We found species differences in the probability of looking at the demonstration: dog puppies were more likely to look than wolf pups and kittens (CMM of looking at demonstration, effect of species: χ^2^_2_ = 54.045; P < 0.001; dog puppies → kittens: exp(β) = 0.311 [0.242; 0.400], z = − 9.12, P < 0.001; dog puppies → wolf pups: exp(β) = 0.418 [0.288; 0.606], z =− 4.60, P < 0.001; Fig. 3a).

Subjects were more likely to look at the demonstration in the ghost trials than in the experimental trials (effect of condition: χ^2^_2_ = 28.623; P < 0.001; experimental → ghost: exp(β) = 1.979 [1.558; 2.513], z = 5.60, P < 0.001). In addition, we also found a species-specific effect of condition (effect of species x condition interaction: χ^2^_2_ = 19.329; P < 0.001). Since dog puppies almost always looked at the demonstration, irrespective of the condition (in 97.5% of experimental trials and in 94.3% of the ghost trials) this interaction was driven by a more pronounced difference in kittens and wolves than in dogs (experimental → ghost in kittens vs. dog puppies: exp(β) = 2.474 [1.542; 3.967], z = 3.76, P < 0.001; experimental → ghost in wolves vs. dog puppies: exp(β) = 3.972[1.504; 10.488], z =2.78, P = 0.005; Fig. 3b).

The subjects of all species were also less likely to look at the demonstration with trials (effect of trial: χ^2^_2_ = 9.586; P = 0.008; T1→T2: exp(β) = 0.850 [0.697; 1.038], z = − 1.59, P = 0.110; T1→T3: exp(β) = 0.725 [0.591; 0.888], z = − 3.10, P = 0.002).

### Interacting with the object

**Figure 4:**
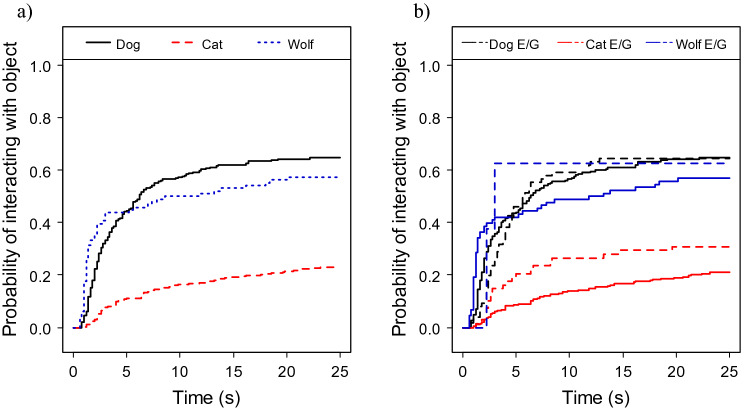
(**a**) Species differences: dog puppies and wolf pups interacted more likely with the object than kittens; (**b**) species-specific effects of condition (experimental condition, E, illustrated with solid, and ghost condition, G, with dotted line) on the probability of interacting with the object in dog puppies, kittens and wolf pups: kittens and, to a lesser extent, also wolf pups (but not dog puppies) interacted with the object more likely in the ghost trials than in the experimental trials.

The three species were also different in the probability of interacting with the object, due to dog puppies and wolf pups being more likely to interact than kittens (CMM of interacting with the object, effect of species: χ^2^_2_ = 30.248; P < 0.001; dog puppies → kittens: exp(β) = 0.204 [0.117; 0.356], z = − 5.58, P < 0.001; dog puppies → wolf pups: exp(β) = 1.113 [0.476; 2.602], z =0.25, P = 0.810; Fig. 4a). Kittens overall interacted only in 30.1% of trials.

We also found that kittens and, to a lesser extent, also wolf pups (but not dog puppies) more likely interacted with the object in the ghost trials than in the experimental trials (CMM of interacting with the object, effect species x condition interaction: χ^2^_2_ = 7.709; P = 0.021; experimental → ghost in kittens vs. dog puppies: exp(β) = 1.965 [0.926; 4.172], z = 1.76, P = 0.079; experimental → ghost in wolf pups vs. dog puppies: exp(β) = 0.325 [0.089; 1.193], z =− 1.69, P = 0.09; Fig. 4b).

All subjects interacted differently over the trials (effect of trial: χ^2^_2_ = 13.278; P = 0.001) and they interacted more likely in the push trials than in the touch trials (effect of touch or push: χ^2^_2_ = 10.007; P = 0.002; T → P: exp(β) = 0.638 [0.483; 0.843], z = − 3.17, P = 0.001), so our final model included both trial number and touch or push. The latter variable, however, had no value for the free exploration trial. Therefore, to see the full effect of demonstration trials as opposed to free exploration trials, we carried out a separate analysis for each species, focusing only on the effect of trial. We found that dogs interacted with the object more in the first trial when there was demonstration compared to the free exploration trial and to in the rest of the trials (CMM of interacting with the object, dog puppies, effect of trial: χ^2^_2_ = 6.678; P < 0.083; T0 → T1: exp(β) = 1.684 [1.089; 2.603], z = 2.35, P = 0.019; T0 → T2: exp(β) = 1.197 [0.762; 1.880], z =0.78, P = 0.430; T0 → T3: exp(β) = 1.136 [0.727; 1.776], z =0.56, P = 0.580). Wolf pups showed a similar trend towards more likely interaction in the first trial (CMM of interacting with the object, wolf pups, effect of trial: χ^2^_2_ = 7.531; P = 0.057; T0 → T1: exp(β) = 3.272 [1.333; 8.031], z = 2.59, P = 0.010; T0 → T2: exp(β) = 2.268 [0.880; 5.848], z =1.69, P = 0.090; T0 → T3: exp(β) = 2.089 [0.850; 5.136], z =1.60, P = 0.11). In kittens there was no significant or trend-like difference between trials (CMM of interacting with the object, kittens, effect of trial: χ^2^_2_ = 3.383; P = 0.336).

### Body part used during free exploration

In the free exploration trials in which the subjects interacted with the object, they almost invariably did so with their nose irrespective of species (GLMM of body part used, effect of intercept: β ± SE = − 1.095 ± 0.384, z = − 2.85, P = 0.004).

### Action matching

**Figure 5:**
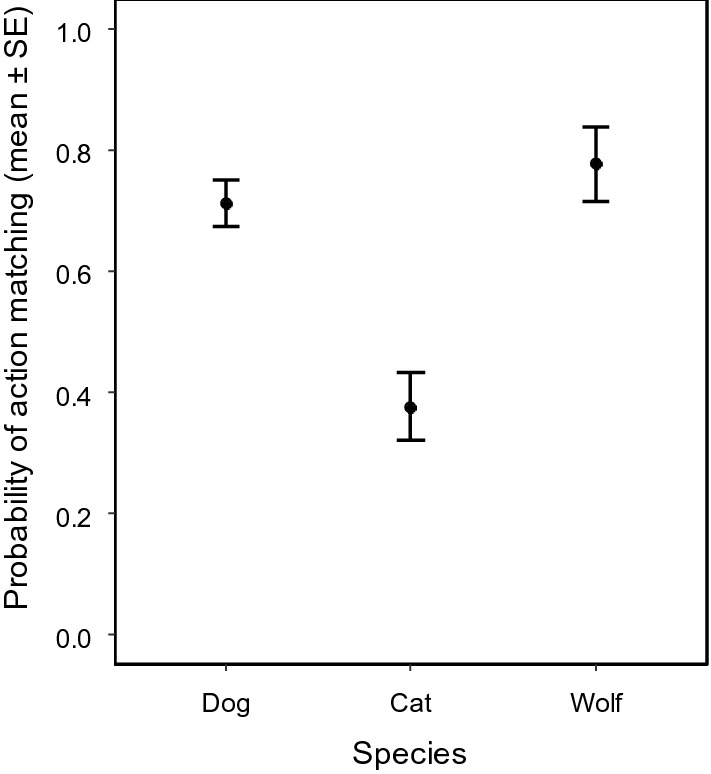
Probability of action matching in dog puppies, kittens and wolf pups. Dog puppies and wolf pups matched their actions to the demonstrated one more often than kittens (GLMM of action matching, effect of species: χ^2^_2_ = 11.931; P = 0.003; dog puppies → kittens: exp(β) = 0.046 [0.005; 0.431], z = − 2.70, P = 0.007; dog puppies → wolf pups: exp(β) = 1.249 [0.081; 19.324], z = 0.16, P = 0.874).

Dog puppies and wolf pups matched their actions to the demonstrated one (nose or hand demonstration) more often than kittens (GLMM of action matching, effect of species: χ^2^_2_ = 11.931; P = 0.003; dog puppies → kittens: exp(β) = 0.046 [0.005; 0.431], z = − 2.70, P = 0.007; dog puppies → wolf pups: exp(β) = 1.249 [0.081; 19.324], z = 0.16, P = 0.874; Fig. 5).

### Probability of paw use based on demonstrated action

Considering all trials, the probability of paw use was different across species (GLMM of paw use, effect of species: χ^2^_2_ = 15.916, P < 0.001; dog puppies → kittens: exp(β) = 0.013 [0.001; 0.143], z = − 3.56, P < 0.001; dog puppies → wolf pups: exp(β) = 3.475 [0.149; 81.060], z = 0.78, P = 0.438). We also found differences in paw use based on the body part used for demonstration (effect of demonstrated action: χ^2^_2_ = 4.970; P = 0.026; nose demonstration → hand demonstration: exp(β) = 6.320 [1.132; 35.281], z = 2.10, P = 0.036).

**Figure 6:**
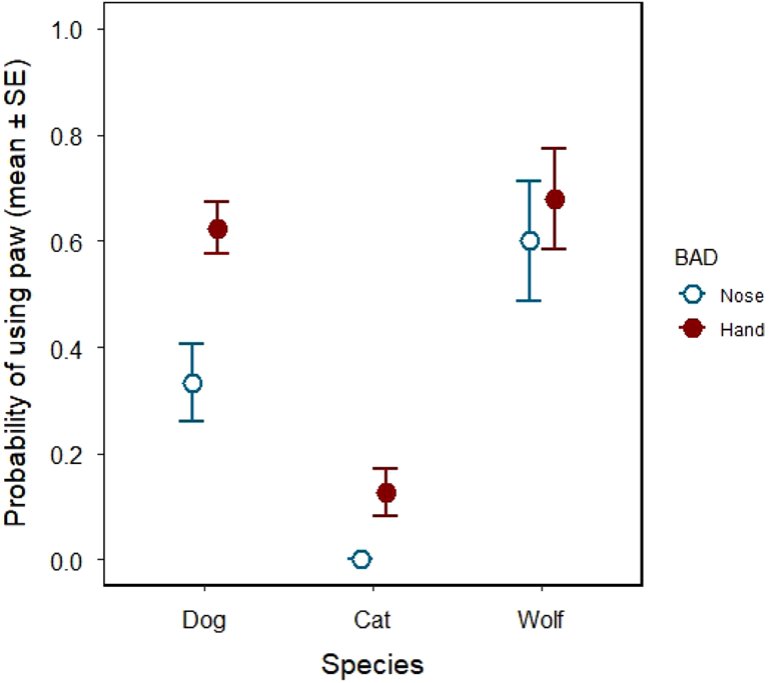
Probability of using paw in dog puppies, kittens and wolf pups, based on the body action of the demonstration (‘BAD’: nose or hand) by the human. Dog puppies used their paw to interact with the object more likely in those trials in which the human demonstration was shown by hand, compared to those in which it was shown by nose.

Our analysis focusing on each species separately revealed species-specific differences in probability of paw use based on demonstration. Dog puppies used their paw to interact with the object significantly more likely in those trials in which the human demonstration was shown by hand, compared to those in which it was shown by nose (GLMM of paw action, effect of demonstrated body action: χ^2^_1_ = 5.696; P = 0.017; nose demonstration → hand demonstration: β ± SE = 2.899 ± 1.408, z = 2.06, P = 0.039; Fig. 6). For kittens, data could not be statistically analysed partly because of the low frequency of interaction with the object and partly because paw actions never occurred when nose actions where demonstrated. They used their paw only in seven trials; in all these seven trials, the demonstration was done by hand. In contrast, from the 22 trials in which the demonstration was done by nose, kittens performed no paw action at all. In wolf pups, we did not find significant probability of using paw when hand actions were demonstrated (effect of demonstrated body action: χ^2^_1_ = 0.189; P = 0.664) (Fig. 6).

## Discussion

The main aim of the study was to test whether dog puppies, kittens and wolf pups show a spontaneous tendency to produce matching actions of a human demonstrator in a novel situation – i.e. in presence of a novel object - in the absence of food reward and previous training. Overall, dog puppies showed the greatest tendency to attend to the human experimenter and were more likely to use their paw when the demonstration was performed with hand, compared to when it was performed using nose. Kittens were the species least affected by the human demonstrations.

A pre-requisite for social learning is paying attention to the demonstration. We found that dog puppies were more likely to look at the demonstration than kittens and wolves. This is consistent with results found in a different experimental paradigm, the impossible task, in older dog puppies and wolf pups^35^, and on adult dogs and cats^36^. In both studies, dogs tended to look at the human and back to the hidden food when they were unable to get the reward, whereas wolves and cats seldom did so.

Interestingly, we also found that kittens and wolf pups – but not dog puppies – looked more likely at the demonstration in the ghost trials, when there was no demonstrator, compared to when the human experimenter performed the demonstration. This suggests that, in contrast to dog puppies, wolf pups’ and kittens’ attention was more attracted by the movement of the object alone than by the human demonstrator.

We also found a decreased tendency of all species to look at the demonstration with increasing trials. This could be due to habituation, as the same demonstrations were repeated 3 times, with no relevant consequence for the subjects (food or other extrinsic motivators were not present).

The increased probability of interaction with the object of dog puppies in the trials with demonstration, compared to the free exploration trial, may be explained by stimulus enhancement^30^. A similar trend was also found in wolf pups, but not in kittens. This suggests that they are less prone to increase their attention and motivation to interact with an object that has just been manipulated by a human demonstrator.

As opposed to dog puppies and, to some extent, to wolf pups, kittens interacted very few times with the object, and the human demonstration did not increase their tendency to interact. Kittens and, to a lesser extent, wolf pups, interacted with the object more in trials that did not include a human demonstration at all (ghost trials). Thus, kittens showed little interest in the human experimenter’s demonstration, but were more attracted by the object’s movement alone.

In the free exploration trials, when the subjects could freely interact with the object in the absence of a demonstration, those who interacted with the object almost invariably did so with their nose. Thus, our two-action method revealed that, by reproducing hand/paw actions, dog puppies (but not wolf pups) tended to perform a behaviour that matched the demonstrated one and also differed from their own spontaneous way of interacting with the object.

Since the human demonstration did not increase kittens’ tendency to interact with the object, kittens interacted with the object in too few trials to provide conclusive results on action matching.

Overall, these results support that dogs show a higher social orientation toward humans and a stronger tendency to match their actions, compared to the other tested species.

While most studies on social learning in dogs involve the use of food (or toys) as rewards, a few studies on behavioral mimicry showed that dogs synchronize some aspects of their behaviour with humans’ (e.g.^37,38^). Our results show that dog puppies tend to reproduce a non-preferred action demonstrated by a human. This tendency is spontaneous – i.e. is not elicited by specific extrinsic motivators such as food.

Our results are also consistent with the results of Range et al.^39^ and Huber et al.^13^, suggesting a tendency for matching actions in dogs. Importantly, our study demonstrates that this tendency in puppies is not elicited by food rewards, but occurs spontaneously. Thus, this action matching tendency may mainly serve the purpose of maintenance of social cohesion and affiliation^4,5^.

Dog puppies’ tendency to match the demonstrated action is in apparent contrast with Fugazza et al.^10^, in which dog puppies learned from humans and conspecifics how to obtain food from a puzzle box, but did not show evidence of action matching. However, this may rather reflect dogs’ tendency to engage in imitation or emulation depending on the presence of a relevant goal in the demonstration (i.e. obtaining food)^40^. Since in Fugazza et al.^10^ the modeled action was aimed at reaching food, the puppies were most likely focusing on this goal, rather than on the means to reach it, and thus they got engaged in goal emulation.

In accordance to this interpretation, in a recent study on over-imitation in adult dogs^41^, only a small minority of dogs over-imitated an irrelevant action demonstrated after attracting the dogs attention with food. Furthermore, the tendency to over-imitate the owners’ actions did not seem to be related to behavioural variables linked to the relationship with him/her. The different age of the dogs tested in Huber et al.^41^ compared to ours is a possible explanation for the apparently contrasting results. Alternatively (or additionally), it is possible that the presence of food both before and at the end of the demonstration in Huber et al.^41^, facilitated goal emulation, rather than matching of the demonstrator’s actions, independently from the relationship the dogs had with their owners.

In the present study, no food-directed actions were shown, and puppies tended to match their actions to the human demonstration. This further suggests that, while the absence of food facilitates spontaneous action matching, its presence in social contexts may distract puppies from social learning of the actions themselves.

Overall, dog puppies’ and, to some extent, wolf pups’ (but not kittens’) increased tendency to interact with the objects in trials that included a demonstration, and their tendency to match the demonstrated actions support the hypothesis that the inherent sociality of the species plays a role in shaping their social learning tendencies. Additionally, the greater tendency of dog puppies, but not socialized wolf pups, to match the non-preferred actions demonstrated by the human in the absence of reward, supports the role of domestication (e.g.^35^). This role, however, seem to depend on the specific domestication history of the species, since kittens, a species with a rather different domestication history, showed the least predisposition to attend to human demonstrations. This suggests that dogs’ unique domestication history may have led to an increased predisposition to attend to, and learn from human models, and/or a decreased tendency to rely on species-specific actions in social contexts with humans (e.g.^9,10,21^).

We suggest that this tendency of dog puppies, if enhanced further and/or not suppressed, may lead to increased behavioural conformation as adults. These results are also important from an applied perspective, and may lead to puppy-training methods that are less reliant on the use of food rewards, and rather exploit and enhance their natural action matching tendency. This could lead to the development of novel ways of teaching new tasks to puppies and adult dogs, relying on their spontaneous tendency for action matching.

### Limitations

We cannot exclude the potential effect of different developmental speeds in the three species. However, our results are also in line with previous studies on both adult and young subjects (e.g.^10,35,36,39^).

Although all the subjects tested in this study were raised and lived in human families and had extensive experience of living with humans, only the wolves were hand-raised. However, we argue that the more extended and earlier experience of living in a human family provided more opportunities to learn socially from them. Yet, overall, the tendency of reproducing human actions was more pronounced in dog puppies than in wolf pups.

While differences between hand-raised dogs and hand-raised wolves are evident from previous studies^35,42^ and are in line with our results, we cannot exclude that kittens, if bottle fed and raised by humans since very early, would have behaved differently.

Additionally, the raising of kittens and dog puppies in human families might have been different to some extent. For example, although owners of dog puppies are typically advised not to take them out until they complete their vaccinations, they might have still taken the puppies out more often than our kitten owners, or than owners that keep their kittens outdoor.

Studies including socialized wolves typically rely on low sample sizes (e.g.^9,35,43^) and related individuals, due to feasibility reasons. It has to be emphasized that the conclusions of such studies should be taken cautiously with regard to generalizability of the results to the species. In this respect, the present study is no exception.

## Conclusions

Our results show that dog puppies, but not hand-raised wolf pups, tend to match a non-preferred human action shown by humans, in the absence of food rewards. Kittens and wolf pups tend to pay less attention to human demonstrations and kittens seem to be overall less influenced by those. These results support the role of both, the inherent sociality of the species, and the effect of domestication, and also provide the basis to devise dog-training methods that rely on this predisposition, rather than on the use of food reward. Future research should further investigate whether including food in social learning situations may (negatively) affect dog puppies’ tendency to match human actions and whether kittens may show some tendencies to learn socially from humans if raised differently or tested in different situations.

## Data Availability

The datasets used and/or analysed during the current study are available from the corresponding author on reasonable request.
